# An experience- and preference-based EQ-5D-3L value set derived using 18 months of longitudinal data in patients who sustained a fracture: results from the ICUROS

**DOI:** 10.1007/s11136-022-03303-y

**Published:** 2022-12-10

**Authors:** Axel Svedbom, Fredrik Borgstöm, Emma Hernlund, Vidmantas Alekna, Maria Luisa Bianchi, Patricia Clark, Manuel Diaz-Curiel, Hans Peter Dimai, Mikk Jürisson, Olga Lesnyak, Eugene McCloskey, Kerrie M. Sanders, Stuart Silverman, Marija Tamulaitiene, Thierry Thomas, Anna N. A. Tosteson, Bengt Jönsson, John A. Kanis

**Affiliations:** 1 ICON, Stockholm, Sweden; 2 LIME/MMC, Karolinska Institustet, Stockholm, Sweden; 3 Faculty of Medicine, Vilnius University, Vilnius, Lithuania; 4 Bone Metabolism Unit, Istituto Auxologico Italiano IRCCS, Milan, Italy; 5 Clinical Epidemiology Unit, Hospital Infantil Federico Gómez and Faculty of Medicine UNAM, Ciudad de Mexico, Mexico; 6 Servicio de Medicina Interna/Enfermedades Metabolicas Oseas, Fundacion Jimenez Diaz, Madrid, Spain; 7 Catedra de Enfermedades Metabolicas Óseas, Universidad Autonoma, Madrid, Spain; 8 Division of Endocrinology and Diabetology, Department of Internal Medicine, Medical University of Graz, Graz, Austria; 9 Faculty of Medicine, University of Tartu, Tartu, Estonia; 10 North-West State Medical University Named After I.I.Mechnikov, St.Petersburg, Russian Federation; 11 Academic Unit of Bone Metabolism, Metabolic Bone and Centre for Integrated Research in Musculoskeletal Ageing University of Sheffield, Sheffield, UK; 12 Department of Clinical Medicine, Western Health and Sunshine Campus Melbourne University, Victoria, Australia; 13 Cedars-Sinai Medical Center, Los Angeles, USA; 14 Department of Rheumatology, Hôpital Nord, Centre Hospitalier Universitaire (CHU) Saint-Etienne, INSERM U1059, Lyon University, Saint-Etienne, France; 15 The Dartmouth Institute for Health Policy and Clinical Practice, Geisel School of Medicine at Dartmouth, Lebanon, USA; 16 Stockholm School of Economics, Stockholm, Sweden; 17 Centre for Metabolic Bone Diseases, University of Sheffield, Sheffield, UK; 18 Division of Dermatology and Venereology, Department of Medicine, Karolinska Institutet, Stockholm, Sweden

**Keywords:** Osteoporosis, Fracture, Health-Related Quality of Life, Health Utility

## Abstract

**Introduction:**

EQ-5D-3L preference-based value sets are predominately based on hypothetical health states and derived in cross-sectional settings. Therefore, we derived an experience-based value set from a prospective observational study.

**Methods:**

The International Costs and Utilities Related to Osteoporotic fractures Study (ICUROS) was a multinational study on fragility fractures, prospectively collecting EQ-5D-3L and Time trade-off (TTO) within two weeks after fracture (including pre-fracture recall), and at 4, 12, and 18 months thereafter. We derived an EQ-5D-3L value set by regressing the TTO values on the ten impairment levels in the EQ-5D-3L. We explored the potential for response shift and whether preferences for domains vary systematically with prior impairment in that domain. Finally, we compared the value set to 25 other EQ-5D-3L preference-based value sets.

**Results:**

TTO data were available for 12,954 EQ-5D-3L health states in 4683 patients. All coefficients in the value set had the expected sign, were statistically significant, and increased monotonically with severity of impairment. We found evidence for response shift in mobility, self-care, and usual activities. The value set had good agreement with the only other experience- and preference-based value set, but poor agreement with all hypothetical value sets.

**Conclusions:**

We present an experience- and preference-based value set with high face validity. The study indicates that response shift may be important to account for when deriving value sets. Furthermore, the study suggests that perspective (experienced versus hypothetical) is more important than country setting or demographics for valuation of EQ-5D-3L health states.

**Supplementary Information:**

The online version contains supplementary material available at 10.1007/s11136-022-03303-y.

## Introduction

The EQ-5D 3L descriptive system is generic multi-attribute utility instrument for measuring health-related quality of life (HRQoL). The instrument comprises five dimensions (mobility, self-care, usual activities, pain/discomfort, and anxiety/depression) with three levels equivalent to “no problems,” “some problems,” and “severe problems,” resulting in a total of 243 (3^5^) health states [1]. The health states can be linked to a ‘value set,’ to derive a summary ‘index value’ (‘utility’), anchored at 1 (perfect health) and 0 (death) [2]. Value sets are derived in stand-alone studies where participants rate EQ-5D health states [2]. The health states and ratings of the participants are combined in regression models where the resulting coefficients constitute the value set [2]. In studies that develop value sets, ratings of health states are done on a visual analog scale (VAS) or using a preference-based time trade-off (TTO) method [1]. It has been argued that preference-based methods such as the TTO are better suited to derive HSUVs to inform decisions on research allocation [3]. One reason is that the TTO involves a sacrifice which is congenial to decisions on resource allocation under conditions of scarcity [4].

There are two main considerations when eliciting values for generic health states: (i) Whose values should be elicited, and (ii) what should be valued [5, 6]. The first consideration pertains to whether the ratings should reflect the preferences of patients or those of the public, and the second whether the participants should rate hypothetical health states or health states they have experienced. In general, individuals with first-hand experience of impaired health states tend to value these health states higher than individuals without such experience [6]. This tendency is especially strong in individuals who value their own health [6]. Historically, most HTA agencies have preferred utilities derived from the general public using hypothetical health [6] but utilities derived from patients and those who have experienced the health states are gaining more support [7]; parallel use of HSUVs derived using patient and public preferences have been advocated [8]; and the second panel of cost-effectiveness identified the relationship between rating of public and patient preferences a research priority [9].

Most value sets—especially those using TTO—reflect public preferences for hypothetical health states [10].To the best of our knowledge, only one EQ-5D 3L preference-based value set using experience-based health exist: A TTO value set derived using data from a postal survey of the general public in Sweden [11].

Furthermore, as far as we are aware, all current value set were derived in cross-sectional settings (during one session for each participant) and therefore it is implicitly assumed that preferences for health states remain constant as health changes. However, the process of adaptation may change patients’ preferences for health—a concept known as response shift. Several theoretical frameworks for defining and understanding response shift exist [12]. In HRQoL research, a popular framework posits that ‘response shifts’ are triggered by changes (catalysts) to health states, leading to changes in internal standards (recalibration), values (reprioritization), or meaning (reconceptualization) of the measured health constructs [13]. Reprioritization may be of special importance for multidimensional HRQoL instruments such as the EQ-5D as reprioritization relates to change in relative importance of health dimensions [14]. It would also be valuable to better understand whether preferences for domains vary systematically with prior impairment in that domain.

Deriving an EQ-5D 3L value set using longitudinal data on patients who experience shifts in health state over time would provide an alternative source for HSUVs and allow for exploring systematic differences in patients’ valuation of their currently experienced health state (“own health”) over time. To these ends, we analyzed data from the prospective International Costs and Utilities after Osteoporotic fracture Study (ICUROS) with three objectives: (i) to derive a value set using patients’ preferences for the own current health (the “ICUROS value set”); (ii) to explore the potential impact of response shift and prior health impairment on value sets; and (iii) to compare and contextualize the ICUROS value set with existing EQ-5D-3L preference-based value sets.

## Methods

### Participants

The ICUROS was conducted under the auspices of the International Osteoporosis Foundation and enrolled patients from 11 countries (Australia, Austria, Estonia, France, Italy, Lithuania, Mexico, Russia, Spain, the UK, and the USA), albeit the US arm of the study did not include the TTO question and was not included in the study at hand.

The ICUROS enrolled patients who had recently (within two weeks) had their first healthcare contact for a low-energy fracture. Patients had to be aged 50 years or more, live in their own home prior to the fracture, having sustained a fracture that was not caused by a co-morbidity (e.g., cancer), and judged to be capable of answering the patient-related questionnaire. Patients with any type of low-energy fractures could be enrolled, but a substantial majority of the patients had hip fracture, distal forearm fracture, or vertebral compression fracture. Vertebral compression fractures were confirmed by x-ray examination. Health-Related Quality of Life was elicited during scheduled contacts with patients at enrollment (pre-fracture recall [phase 0] and current [phase 1]), and at 4 months (phase 2), 12 months (phase 3), and 18 months [phase 4] after enrollment. Thus, during the enrollment visit that took place within two weeks of fracture, patients were asked to rate both their own current HRQoL (phase 1) and the HRQoL they experienced before the fracture (phase 0). In all phases the patients rated their own current HRQoL using the EQ-5D 3L and the TTO instrument. The study design has been described in more detail elsewhere [15, 16].

During enrollment the patient populated a clinical report form (CRF) guided by a study nurse. Follow-up interviews were conducted over the telephone.

### Outcomes and variables

The main variables of interest in this study were the EQ-5D-3L descriptive system, the EQ-VAS, and a TTO task. Those were collected for each phase. Information on country, fracture site, age at fracture, sex, income (patient reported), and education (patient reported) were obtained at study enrollment. The official EQ-5D-3L and EQ-VAS versions of the 10 participating countries were used. The TTO question and visual aid are presented in the Online Supplement.

Education was reported in five categories and patients reported the highest level achieved: Primary, secondary, professional diploma, and university. Income had three categories: low, middle, or high.

### Value set

We derived the value set using a regression model on patient-level data. The outcome variable in the regression models was TTO at each visit and independent variables comprised dichotomous variables for impairment in each EQ-5D dimension. We use the following abbreviations to facilitate readability: MO for mobility, UA for usual activities, SC for self-care, PD for pain/discomfort, and AD for anxiety/depression. Furthermore, we use “2 “ to denote “some problems” and “3” to denote “severe problems.” For example, MO2 denotes “some problems” in the mobility dimension and SC3 denotes “severe problems” in the self-care dimension. The model is presented in more detail in the online supplement.

### Response shift

The ICUROS study was not originally designed to measures response shift but the data were collected for other purposes. Therefore, the study at hand aims to evaluate response shift from secondary data and the analyses were informed by guidelines for this specific study type [14]. We focused on response shift from the measurement time-point immediately after fracture to subsequent time points. The reason is two-fold: Changes to health domains are likely most rapid in the first months after fracture, making the potential for response shift larger during this time-period. Less drastic changes to health may not produce important changes in outcomes on the group level and therefore no measurable response shift [14]. Secondly, it has been argued that respondents rating hypothetical health states imagine themselves transitioning to the health state in question and therefore may value it differently than if they would have been accustomed to or better informed about the health state in question [7]. Exploring whether response shift occurs after an initial actual transition in health may help to support or reject this notion.

### Valuation of domains with prior impairment

We explored whether valuation of specific domains varied systematically with prior impairment in that domain by comparing the health state valuations of those with impaired health before fracture and those without impaired health before fracture for each dimension.

To contextualize the impact of response shift and prior impairment, we compared the estimates to the minimally important difference for EQ-5D 3L, which has been estimated at 0.074 for the UK Measurement and Valuation of Health (MVH) value set [17].

### Comparisons of value sets

We compared the value set derived using the ICUROS to all other value sets derived using TTO available in the R package EQ-5D [18] by estimating the intraclass correlation coefficients (ICC), a measure of agreement between two ratings, using all 243 health states. To contextualize the agreement, we also estimated the ICCs for ICUROS value set, the Swedish TTO experience-based value set [11], and the UK TTO hypothetical MVH value set [19] to the value sets available in the R package EQ-5D.

### Statistical analysis

Patient characteristics were described using frequencies and percentages for dichotomous variables and median and interquartile ranges for continuous variables.

In the regression models, we excluded data for the pre-fracture recall as outcome given that they are not based on the patient’s current experience. However, pre-fracture recall was used for stratification of patients in the analysis of impact of prior impairment. We excluded phase 1 data for patients who reported higher TTO at enrollment than before the fracture given that this indicated that patients may have misunderstood the TTO task. We also excluded data from contacts where patients reported (i) “severe problems” with mobility but “no problems” with usual activities or self-care, and “severe problems” in usual activities but “no problems” with self-care. The rationale for this exclusion is that these combinations of impairment may be considered inconsistent with each other and could therefore indicate data entry errors: either on part of the patient or the study nurse. Given that single patients could contribute with multiple observations and that patients may be more similar within hospitals, countries, and phases than between hospitals, countries, and phases, we modeled random effects [20] for patients, hospitals, countries, and phases.

We derived the value set by fitting a linear mixed effects regression model with ten dichotomous variables—one for each level of impairment in the five EQ-5D dimension—on TTO. The regression model is presented in the online supplement.

We assessed the model using split sample validation. Observations were assigned with equal probability to the estimation and validation samples. We derived the coefficients in the estimation sample and estimated the mean absolute error, the root mean square error, and the Pearson correlation coefficient by comparing predicted and observed TTO values in the validation sample.

To explore response shift, we followed the approach suggested by Lowy and Bernhard (2004) [21] and fit a mixed effects regression model with an interaction for the response shift time-point. Consequently, we entered interaction terms for phase 1 (the contact immediately after fracture) and each of the ten variables in the mixed effects regression model used to derive the value set. Hence, the model included 22 variables: ten for impairments, one categorical variable for phase 1, a constant, and the product between the categorical variable for phase 1 and the ten impairment variables. The coefficient for an interaction can be interpreted an estimate of the mean HSUV difference of experiencing a particular impairment in Phase 1 compared to subsequent phases. In essence, this approach compared a predicted value to an observed value, with the difference attributed to a response shift. The regression model is presented in the online supplement.

To explore the impact of prior impairment, we entered interaction terms for any impairment prior to fracture in each of the five dimensions with the corresponding impairment variables in the mixed effects regression model used to derive the value set, excluding phase 1. Hence, the model included 26 variables: ten for impairments, five categorical variables for any impairment prior to fracture, and ten terms for the product between the categorical variable for impairment in each dimension prior to fracture and the categorical variables for “some” and “severe” impairment in each dimension, and a constant. The coefficient for an interaction is an estimate of the mean HSUV difference for a specific impairment in patients who had impaired health in that dimension prior to fracture compared to those who did not have impaired health in that dimension prior to fracture. The coefficient may be interpreted as the impact of prior impairment in a domain on the valuation of that domain, all else equal. The regression model is presented in the online supplement.

We described the agreement between value sets by estimating individual absolute-agreement ICCs between value sets across all 243 EQ-5D health states using two-way random effect models. We categorized agreement for ICC point estimates between value sets below 0.40 as poor, between 0.40 and 0.59 as fair, between 0.60 and 0.74 as good, and above 0.75 as excellent [22, 23].

The HSUVs for all value sets except the ICUROS value set were obtained using the package EQ-5D in R version 3.6.2. All other analyses were done in Stata version 16.2 (StataCorp LP, College Station, TX, USA).

## Results

### Patient selection

Among the 6298 patients enrolled in ICUROS with data after fracture, 6295 patients reported data for 20,220 experienced health states. After having removed patient without TTO data, implausible EQ-5D health states and TTO scores 12,954 health states in 4683 patients were eligible for analysis (Online Resource Figure S2).

### Patient characteristics

Patient characteristics are presented in Table 1. Patients were predominately female (3,789/4,683; 81%) and the median (IQR) age was 71 years (61–79). The number of patients per country ranged from 145 (3%) for Italy to 1047 (22%) for Russia and hip fracture was the most common fracture site (1840, 39%). Recalled median (IQR) TTO and EQ-VAS prior to fracture were 1.00 [0.80–1.00] and 0.80 [0.68–0.98], respectively.

**Table 1:** Patient characteristics at enrollment

Characteristic	
Patients	4683
Female sex *n* (%)	3789 (80.9%)
Age at fracture median (IQR)	71 (61, 79)
Fracture site *n* (%)	1840 (39.3%)
Hip	
Distal forearm	1322 (28.2%)
Vertebral	650 (13.9%)
Humeral	398 (8.5%)
Ankle	394 (8.4%)
Other	79 (1.7%)
TTO prior to fracture, median (IQR)	1.00 (0.80, 1.00)
EQ-VAS prior to fracture, median (IQR)	0.80 (0.68, 0.90)
Country *n* (%)	
Austria	716 (15.3%)
Australia	622 (13.3%)
Estonia	197 (4.2%)
Spain	343 (7.3%)
France	319 (6.8%)
Italy	145 (3.1%)
Lithuania	609 (13.0%)
Mexico	353 (7.5%)
Russia	1047 (22.4%)
United Kingdom	332 (7.1%)
Level of income *n* (%)	
Low	1706 (37.0%)
Medium	2254 (48.9%)
High	475 (10.3%)
Prefer not to answer/missing	248 (5.3%)
Highest level of education	
Primary school	1369 (29.6%)
Secondary school	1957 (42.3%)
University	1093 (23.6%)
Other	208 (4.5%)
Missing	56 (1.2%)

### Experienced health states and reported TTO values

The number of observations for different levels of impairment in the five dimensions ranged from 1,108 to 8,175 (Table 2) across all observations. When stratifying health states between Phase 1 and subsequent phases, the most infrequent impairments were “No impairment” in usual activities in Phase 1 and severe mobility impairment during subsequent phases, each observed 194 times. Among the 240 possible health states (three health states excluded), 128 were experienced at least once, 100 at least five times, and 85 at least ten times. The TTO values ranged from 0 (negative values not possible) to 1.00 and the median [IQR] TTO was 0.90 [0.55 to 1.00].

**Table 2:** Distribution of severity of impairment by dimension and by all, and acute (phase 1) and non-acute (phases 1, 2, and 3) phases

	Mobility	Self-care	Usual activities	Pain and discomfort	Anxiety/depression
All phases					
No problem *n* (%)	6101 (47)	7081 (55)	5340 (41)	4730 (37)	8175 (63)
Some problem *n* (%)	5225 (40)	4087 (32)	5189 (40)	6687 (52)	3671 (28)
Severe problem *n* (%)	1628 (13)	1786 (14)	2425 (19)	1537 (12)	1108 (9)
Acute (Phase 1)					
No problem *n* (%)	899 (23)	369 (10)	194 (5)	396 (10)	1568 (41)
Some problem *n* (%)	1520 (39)	1975 (51)	1660 (43)	2381 (62)	1582 (41)
Severe problem *n* (%)	1434 (37)	1509 (39)	1999 (52)	1076 (28)	703 (18)
Non-acute (Phases, 2,3, 4)					
No problem *n* (%)	5202 (57)	6712 (74)	5146 (57)	4334 (48)	6607 (73)
Some problem *n* (%)	3705 (41)	2112 (23)	3529 (39)	4306 (47)	2089 (23)
Severe problem *n* (%)	194 (2)	277 (3)	426 (5)	461 (5)	405 (4)

### Value set

The results from the mixed effects regression model used to derive the value set are presented in Table 3. All coefficients had the expected sign, were statistically significant, and increased monotonically with severity of impairment. The decrements ranged from − 0.019 (95% CI: − 0.029; − 0.008) for “some problems” in pain and discomfort to − 0.135 (95% CI: − 0.152; − 0.117) for “severe problems” in anxiety/depression. The value set ranged from 0.932 to 0.446.

**Table 3:** Coefficients from the linear mixed effects regression model of time trade-off (TTO) values on EQ-5D-3L dimensions

Variable	Mean (95% CI)	*p* value
MO2	− 0.033 (− 0.043; − 0.022)	< 0.001
MO3	− 0.055 (− 0.076;− 0.034)	< 0.001
SC2	− 0.111 (− 0.123;− 0.099)	< 0.001
SC3	− 0.112 (− 0.136;− 0.089)	< 0.001
UA2	− 0.023 (− 0.035;− 0.011)	< 0.001
UA3	− 0.076 (− 0.098;− 0.055)	< 0.001
PD2	− 0.019 (− 0.029;− 0.008)	< 0.001
PD3	− 0.107 (− 0.124;− 0.090)	< 0.001
AD2	− 0.058 (− 0.068;− 0.047)	< 0.001
AD3	− 0.135 (− 0.152;− 0.117)	< 0.001
Constant	0.932 (0.88;0.983)	< 0.001
Prob > F	< 0.001	
AIC	1270	

*MO* stands for mobility, *UA* for usual activities, *SC* for self-care, *PD* for pain/discomfort, and *AD* for anxiety/depression; “2 “ denote “some problems” and “3” to denote “severe problems” in the dimensions

### Validation of value set

**Fig. 1:**
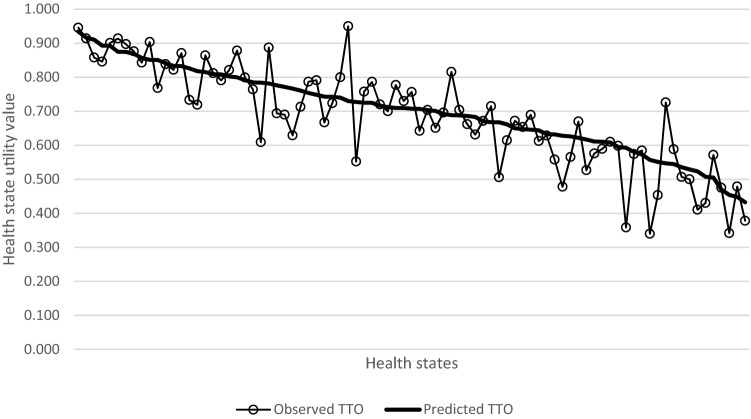
Line plot of mean predicted time trade-off (TTO) values and mean observed TTO values for health states with at least ten observations in the validation set (sorted by predicted TTO value). The health states are ordered from highest to lowest predictive value

The MAE, RMSE, and Pearson correlation coefficients between predicted and observed values in the split sample validation were estimated at 0.178, 0.238, and 0.514, respectively. Predicted and observed TTO for health states with at least ten observations are presented in Fig. 1.

### Response shift

**Fig. 2:**
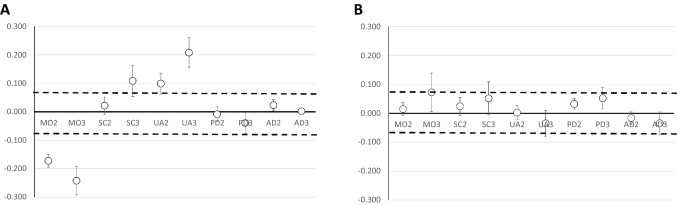
Panel A shows the interaction coefficients and 95% confidence intervals between each level of impairment for the five dimensions of the EQ-5D and Phase 1. Positive values indicate a potential negative response shift with time, i.e., that patients value impairments more with time, whereas negative values indicate a negative response shift, i.e., that patients value impairments less with time. Panel B shows the interaction coefficients and 95% confidence intervals between each level of impairment for the five dimensions of the EQ-5D and any impairment in the same dimension prior to fracture. The dotted lines indicate a clinically significant change

The coefficients for the interactions between phase 1 and MO2, MO3, and PD3 were negative and statistically significant (Fig. 2A), indicating that these impairments had a stronger impact in phase 1 compared to subsequent phases. On the other hand, the coefficients for the interactions between phase 1 and SC3, UA2, UA3, and AD3 were positive and statistically significant (Fig. 2A), indicating that these impairments had a weaker impact in phase 1 compared to subsequent phases. The point estimates of the coefficients for the interaction terms for MO2, MO3, UA2, UA3, and SC3 exceeded the conventional threshold for clinical significance of 0.074. The full regression model for the analysis of response shift is presented in Online Supplement.

### Valuation of domains with prior impairment

The coefficients for the interactions between any impairment in the relevant dimension prior to fracture and MO3, PD2, and PD3 were statistically significant and positive (Fig. 2B), implying that prior impairment may attenuate the effect of these impairments. All the point estimates for the interaction coefficients fell below the conventional threshold for clinical significance of 0.074 (Fig. 2B). The full regression model for the analysis of impact of prior impairment is presented in Online Resource Table S3.

### Comparisons of value sets

The ICCs between the ICUROS and the Swedish experience-based TTO value set was estimated at 0.74 (95% CI: 0.48–0.85), considered good agreement. The agreement between ICUROS and all other value sets were poor. The agreement between the Swedish and the other value sets was fair for four countries and poor for 21 countries. For the UK MVH value set, agreement was considered excellent for 12 value sets, good for 5, fair for 6, and poor for 2. Please see Online Supplement for the ICCs of individual comparisons.

## Discussion

### Principal findings

Using longitudinal data on patients with a recent fragility fracture, we derived a preference- and experience-based EQ-5D-3L value set with high face validity: All coefficients had the expected sign, were statistically significant, and increased monotonically with severity of impairment. Furthermore, we found that response shift may have a substantial effect on the valuation of health states.

### Strengths and weaknesses

To the best of our knowledge this is the first ever preference- and experience-based EQ-5D-3L value set based on a longitudinal data. The main strengths of the study are its prospective design and inclusion of patients expected to transition through different health states during the study, enabling us to address two key challenges when deriving own health-based value sets: Few observations with severe impairments and complete lack of training for the participants [6]. Furthermore, the longitudinal design also allowed for the exploration of response shift in a way that is not possible in cross-sectional studies.

One key weakness of the study is its generalizability. Firstly, the study is based on convenience sampling of patients who had sustained a fragility fracture and the sample is therefore not representative of the general population, or even patients who experience the given health states. Secondly, a substantial proportion of observations with complete EQ-5D-3L data did not have TTO data, indicating further selection bias. Thirdly, the analysis exploring response shift demonstrated that the valuation of health states differed substantially between phase 1 and subsequent phases; the composition of acute and more chronic health states is a function of study design rather than distribution of chronic and acute health states in the population, further limiting generalizability. Finally, the participants came from eleven countries and the study results are not applicable to any specific country. Nevertheless, the good—bordering on excellent—ICC between the ICUROS and Swedish experience-based TTO value set [11] suggests that the ICUROS value set may generalize well in practice in situations where an experience- and preference-based value set is required.

Other weaknesses include that the TTO task did not allow for negative values and that respondents had to report a single value without formal deliberation, thereby not adhering to the most common TTO-methodology [7]. A related limitation is that no visual aids were provided to the patients during telephone contacts, this may have complicated the tasks for the respondents. It may also be noted that we did not measure the time respondents took to complete the tasks and therefore cannot verify that they took sufficient time to properly deliberate on the TTO and EQ-5D questions. A limitation of the analyses of response shift is that the study was not primarily designed to observe response shift and therefore lack information that would be important for a more comprehensive understanding of response shift in the context of either EQ-5D value set derivation or osteoporotic fracture. For example, it would be valuable to understand whether the observed response shift is mainly a function of reprioritization or whether reconceptualization and recalibration also play important roles. Furthermore, it is possible that misspecification of the regression model may have produced inaccurate estimates from which response shift is deduced [12].

It should also be noted that the threshold for clinical significance for HSUV used for comparison is based on the UK MVH value set and therefore may not be applicable to the current data.

### Comparison to other studies

The ICC analysis suggest that the ICUROS value set is similar to the Swedish experience-based value set [11], but dissimilar to the other preference-based value sets. In addition, the ICUROS and Swedish value sets are the only value sets with a maximum below one and minimum above zero. In terms of coefficients, the largest differences between the point estimates for the two experience-based value sets were UA2 and SC3 (combined SC2 and SC3 in the Swedish value set) at 0.085 (more negative in the Swedish value set) and 0.078 (more negative in the ICUROS value set), respectively. The Swedish value set had lower mean absolute error and higher Pearson correlation coefficient than the ICUROS value set, potentially reflecting fewer observations in impaired health states—where measurement error appears greater (cf Fig. 1)—in the Swedish value set.

The difference between the coefficients SC2 (− 0.111 (95% CI: − 0.123; − 0.099)) and SC3 (− 0.112 (95% CI: − 0.136; − 0.089)) was very small in the ICUROS value set. For the Swedish TTO value set, the coefficient for SC2 was lower than for SC3, and several other value sets had similar issues with self-care [11]. This phenomenon has been hypothesized to be an artifact of few observations with impaired self-care in the previous studies [11]. However, the distribution of health states used to derive the ICUROS value set (cf Table 2) does not support this notion.

### Implications

The good agreement between the ICUROS and Swedish value sets and the generally poor agreement between these two value sets and value sets based on hypothetical health indicate that the perspective (experienced versus hypothetical health) is more important than country setting or patient population. These findings suggest that it is more important to choose a value set based on preferred perspective (experienced versus hypothetical health) instead of country setting or patient population. Currently, experienced health plays a limited role in HTA for reimbursement decisions [24]. However, the interest for experienced health in HTA is growing [8].

The existence of response shift is well known and theoretical models of the concept exist [12–14]; but to the best of our knowledge, this is the first study that explores the potential impact of response shift on EQ-5D value sets, important topics given the central role of EQ-5D in estimation of HSUV used in HTA to inform reimbursement decisions. The findings demonstrate that participants’ valuation of mobility, self-care, and usual activities differ substantially between the acute and later phases, indicating that response shift may have substantial impact on health state valuation. This result questions the validity of value sets derived using hypothetical health for the valuation of long-term health impairment. Participants in hypothetical health valuation studies frequently value health states with which they have no experience [8]. It is therefore likely that these participants rate the consequences of the health states more similar to patients who have limited experience of the relevant health state than to patients with longer experience of the health state. Therefore, measuring the impact of long term or chronic impairment using hypothetical health may materially overestimate the impact of mobility impairment on HSUVs but underestimate the impact of usual activities and self-care. This finding may also explain some of the observed discrepancy between HSUVs derived using hypothetical health and experienced health: Experienced health generally measures the long-term utility of a health state, whereas hypothetical health generally measures the short-term utility impact of transitioning to that health state.

In terms of cost-effectiveness analysis for HTA, accounting for response shift may alter the relative cost-effectiveness of treatments. The impact likely depends on whether the health effects of a disease are transitory or chronic and the dimensions of health the disease affects. The consequences of fragility fracture—the event used for the analysis in this study—on HRQoL are partly transitory and impacts mobility, self-care, and usual activities. Therefore, it is difficult to determine whether treatment of osteoporosis would become relatively more or less cost-effective if response shift in health state valuation was accounted for.

### Future studies and conclusions

Further studies on own health and preference-based value sets are warranted and may address important questions on generalizability of value sets in different settings. Especially, it would be beneficial to better understand whether there are substantial differences in value sets derived in patients with different diseases. A longitudinal study incorporating EQ-5D, TTO, and instruments specifically developed to explore response shift such as the QOL Appraisal Profile along with objective measures of impairment in the EQ-5D dimensions could provide information on response shift after fracture in general. Further research on systematic differences and their causes between the preferences of the general population and those who have more experience of the health states are warranted. Such information may be important when deriving EQ-5D value sets and choosing HSUVs for economic evaluations. Another interesting area for future research is the reason for the differing ICCs between the value sets.

In conclusion, this study presented an experience- and preference-based value set with high face validity. The study also indicates that response shift may be important to account for when deriving EQ-5D value sets.

## Supplementary Information

Below is the link to the electronic supplementary material.

Supplementary file1 (DOCX 67 KB)

## Data Availability

Due to ethical concerns, supporting data cannot be made openly available.
