# Supplementary material for: An experience- and preference-based EQ-5D-3L value set derived using 18 months of longitudinal data in patients who sustained a fracture: results from the ICUROS
Source: Qual Life Res. 2022 Dec 10;32(4):1199–208. doi: 10.1007/s11136-022-03303-y (PMC10063467; doi:10.1007/s11136-022-03303-y)
Supplement: Supplementary file 1 — Supplementary file1 (DOCX 67 KB) [file 11136_2022_3303_MOESM1_ESM.docx]

**Online Resource**

This online resource has been provided by the authors to give readers additional information about their work.

Supplement to: An Experience and Preference Based EQ-5D-3L Value Set Derived Using 18 Months Longitudinal Data in Patients who Sustained a Fracture: Results from the ICUROS.

**Table of content**

1. Time trade off question and visual aid
2. Patient enrollment and selection
3. Regression model to derive value set
4. Regression model to explore response shift and results
5. Regression model to explore valuation of domains with prior impairment
6. Comparisons between preference- based value sets

**1. Time-trade-off question and visual aid**

At enrollment, the TTO task comprised a horizontal line representing zero to ten years with years marked with numbers and half-years with tick marks along with a time-trade off question (Figure S1). During follow-up telephone interviews, patients were asked the same question, but no visual aid was provided. The TTO value was derived by dividing the number provided during the task by 10.

The following question time-trade off question was asked: “Imagine that your remaining life expectancy is 10 years with your current health status and that you have a choice between two alternatives: Either you will stay in the current health state for 10 years and then die, or you will have full health, but then you will have to give up some years of life. (Please note that this choice is purely hypothetical). You will thus live for 10 years with current health, or for a shorter period of time in full health. Please write down the number of years in full health that you think is of equal value to 10 years in your current health state.” During the enrolment examination the visual aid below was used. Please note that patients could not use negative values, effectively setting the floor for the value set at 0.

Living with current health for 10 years

is equivalent to living in full health for ______ years.

0

1

2

3

4

5

6

7

8

9

10

Years

**Figure S1.** Time-trade-off visual aid used in the study.

**2. Patient enrollment and sample selection**

No or incomplete EQ-5D data: 3 patients and 283 health states

Enrolled patients: 6,298 with 20,503 health states

No TTO data: 1,440 patients and 6,601 health states

Patients with implausible TTO or EQ-5D data: 172 patients and 565 health states

Patients with at least one complete EQ-5D health state: 6,295 patients with 20,220 health states)

Patients with concurrent EQ-5D and TTO data: 4,885 patients and 13,619 health states

Eligible patients with plausible data and at least one TTO and EQ-5D pair: 4,638 and 12,954 health states

**Figure S2.** Sequential patient selection.

**Patient recruitment and comparison of patient characteristics in included and excluded patients**

In total 6,298 patients enrolled in the ICUROS study with a theoretical total of 20,503 elicited health states. Three patients had incomplete or no EQ-5D data; 1,440 had no TTO data; and 172 patients had implausible EQ-5D and TTO data. Therefore, 4,684 patients with 12,954 health states were eligible for analysis (Figure S2). Excluded patients were generally older (mean age: 74 vs 71; p<0.001) and more frequently female (85% vs 81%; p<0.001) than patients who were included in the study.

**3. Regression model to derive value set**

The outcome variable in the regression models was TTO at each visit and independent variables comprised dichotomous variables for impairment in each EQ-5D dimension. The model therefore included 10 independent variables each corresponding to “some problem” or “severe problem” for each of the five EQ-5D dimensions and a constant. We did not include variables intended to capture interactions and non-linearities between impairments in dimensions given the potential for differences between the valuation of health states immediately after fracture (phase 1) and at subsequent contacts (phases 2 to 4) due to response shift. The linear mixed effects regression model is presented below fitted to derive the value set is presented in Equation 1 below.

TTO = MO2 MO3 SC2 SC3 UA2 UA3 PD2 PD3 AD2 AD (Equation 1)

Note: MO stands for mobility, UA for usual activities, SC for self-care, PD for pain/discomfort, and AD for anxiety/depression. “2“ denotes “some problems” and “3” denotes “severe problems”. Furthermore, we do not show intercepts, error terms or coefficients.

**4. Regression model to explore response shift and results**

To explore response shift we entered interaction terms for phase 1 (the contact immediately after fracture) and each of the ten variables in the mixed effects regression model used to derive the value set. The linear mixed effects regression model is presented in Equation 2 below:

TTO= MO2 MO3 SC2 SC3 UA2 UA3 PD2 PD3 AD2 Phase1 Phase1xMO2 Phase1xMO3 Phase1xSC2 Phase1xSC3 Phase1xUA2 Phase1xUA3 Phase1xPD2 Phase1xPD3 Phase1xAD2 Phase1xAD (Equation 2)

The fitted regression model is presented below.

**Table S1.** Results for the regression model fit to explore the impact of response shift.

| **TTO** | **Coefficient** | | **Std Error** | | **Z-value** | | **P-value** | | **95% CI** | |
| --- | --- | --- | --- | --- | --- | --- | --- | --- | --- | --- |
| Phase 1 | -0.045 | 0.018 | | -2.54 | | 0.011 | | -0.080 | | -0.010 |
| MO2 | 0.006 | 0.006 | | 1.07 | | 0.287 | | -0.005 | | 0.018 |
| MO2xPhase 1 | -0.172 | 0.011 | | -15.04 | | <0.001 | | -0.194 | | -0.149 |
| MO3 | 0.047 | 0.022 | | 2.12 | | 0.034 | | 0.004 | | 0.090 |
| MO3xPhase 1 | -0.242 | 0.026 | | -9.40 | | <0.001 | | -0.293 | | -0.192 |
| SC2 | -0.106 | 0.007 | | -14.57 | | <0.001 | | -0.120 | | -0.092 |
| SC2xPhase 1 | 0.022 | 0.015 | | 1.40 | | 0.162 | | -0.009 | | 0.052 |
| SC3 | -0.174 | 0.022 | | -7.90 | | <0.001 | | -0.217 | | -0.131 |
| SC3xPhase 1 | 0.109 | 0.028 | | 3.87 | | <0.001 | | 0.054 | | 0.164 |
| UA2 | -0.038 | 0.007 | | -5.78 | | <0.001 | | -0.051 | | -0.025 |
| UA2xPhase 1 | 0.099 | 0.019 | | 5.21 | | <0.001 | | 0.062 | | 0.136 |
| UA3 | -0.125 | 0.017 | | -7.25 | | <0.001 | | -0.159 | | -0.091 |
| UA3xPhase 1 | 0.208 | 0.027 | | 7.71 | | <0.001 | | 0.155 | | 0.261 |
| PD2 | -0.018 | 0.006 | | -3.07 | | 0.002 | | -0.029 | | -0.006 |
| PD2xPhase 1 | -0.009 | 0.013 | | -0.64 | | 0.523 | | -0.035 | | 0.018 |
| PD3 | -0.084 | 0.013 | | -6.60 | | <0.001 | | -0.109 | | -0.059 |
| PD3xPhase 1 | -0.039 | 0.019 | | -2.07 | | 0.038 | | -0.076 | | -0.002 |
| AD2 | -0.066 | 0.006 | | -10.23 | | <0.001 | | -0.078 | | -0.053 |
| AD2xPhase 1 | 0.023 | 0.010 | | 2.31 | | 0.021 | | 0.004 | | 0.043 |
| AD3 | -0.131 | 0.013 | | -10.02 | | <0.001 | | -0.157 | | -0.106 |
| AD3xPhase 1 | 0.002 | 0.017 | | 0.10 | | 0.921 | | -0.032 | | 0.035 |
| Constant | 0.927 | 0.026 | | 35.13 | | <0.001 | | 0.875 | | 0.978 |

Note: MO stands for mobility, UA for usual activities, SC for self-care, PD for pain/discomfort, and AD for anxiety/depression; “2“ denote “some problems” and “3” to denote “severe problems” in the dimensions. Phase 1 stands for that the measurement was obtained during Phase 1 (within two weeks of fracture). Std stands for standard and CI stands for confidence interval.

**5. Regression model for valuation of domains with prior impairment**

We contrasted the valuation of each level of impairment in the five dimensions using data from phases 2, 3 and 4 in patients who reported “no problems” prior to fracture to the valuation of patients who reported “some problems” or “severe problems” prior to fracture. In this regression model -“2/3P0” designates impairment in the relevant EQ5D-dimension in phase 0 (i.e. prior to fracture). The regression model for valuation of domains with prior impairment is presented in Equation 3 below.

TTO=intercept + MO2 + MO3 + SC2 + SC3 + UA2 + UA3 + PD2 + PD3 + AD2 + AD3 + MO2/3P0 + SC2/3P0 + UA2/3P0 + PD2/3P0 + AD2/3P0 + MO2x MO2/3P0 + MO3x MO2/3P0 + SC2xSC2/3P0 +SC3xSC2/3P0 + UA2xUA2/3P0 + UA2xUA2/3P0 + UA3xUA2/P0 + PD2xPD2/3P0 + PD3x PD2/3P0 + AD2xAD2/3P0 + AD3xAD2/3P0 (Equation 3)

The fitted regression model is presented below.

**Table S2.** Results for the regression model fit to explore valuation of domains with prior impairment

| **TTO** | **Coefficient** | | **Std. Errror** | | **Z-value** | | **P-value** | | **95% CI** | |
| --- | --- | --- | --- | --- | --- | --- | --- | --- | --- | --- |
| MOimpprior | -0.040 | 0.011 | | -3.77 | | <0.001 | | -0.06 | | -0.02 |
| UAimpprior | 0.000 | 0.012 | | 0.04 | | 0.970 | | -0.02 | | 0.02 |
| SCimpprior | -0.059 | 0.015 | | -3.99 | | <0.001 | | -0.09 | | -0.03 |
| PDimpprior | -0.020 | 0.009 | | -2.22 | | 0.026 | | -0.04 | | 0.00 |
| ADimpprior | -0.012 | 0.008 | | -1.46 | | 0.145 | | -0.03 | | 0.00 |
| MO2 | -0.011 | 0.006 | | -1.78 | | 0.074 | | -0.02 | | 0.00 |
| MO2xMOimpprior | 0.015 | 0.011 | | 1.31 | | 0.192 | | -0.01 | | 0.04 |
| MO3 | 0.047 | 0.030 | | 1.59 | | 0.113 | | -0.01 | | 0.11 |
| MO3xMOimpprior | 0.073 | 0.034 | | 2.14 | | 0.033 | | 0.01 | | 0.14 |
| SC2 | -0.052 | 0.007 | | -7.98 | | <0.001 | | -0.06 | | -0.04 |
| SC2xSCimpprior | 0.024 | 0.016 | | 1.51 | | 0.130 | | -0.01 | | 0.05 |
| SC3 | -0.104 | 0.022 | | -4.71 | | <0.001 | | -0.15 | | -0.06 |
| SC3xSCimpprior | 0.052 | 0.029 | | 1.8 | | 0.072 | | 0.00 | | 0.11 |
| UA2 | -0.040 | 0.006 | | -6.84 | | <0.001 | | -0.05 | | -0.03 |
| UA2xUAimpprior | 0.002 | 0.012 | | 0.2 | | 0.841 | | -0.02 | | 0.03 |
| UA3 | -0.120 | 0.018 | | -6.64 | | <0.001 | | -0.15 | | -0.08 |
| UA3xUAimpprior | -0.035 | 0.023 | | -1.51 | | 0.132 | | -0.08 | | 0.01 |
| PD2 | -0.022 | 0.006 | | -3.84 | | <0.001 | | -0.03 | | -0.01 |
| PD2xPDimpprior | 0.033 | 0.009 | | 3.51 | | <0.001 | | 0.01 | | 0.05 |
| PD3 | -0.097 | 0.015 | | -6.63 | | <0.001 | | -0.13 | | -0.07 |
| PD3xPDimpprior | 0.052 | 0.019 | | 2.73 | | 0.006 | | 0.01 | | 0.09 |
| AD2 | -0.036 | 0.006 | | -5.74 | | <0.001 | | -0.05 | | -0.02 |
| AD2xADimpprior | -0.016 | 0.011 | | -1.5 | | 0.133 | | -0.04 | | 0.00 |
| AD3 | -0.093 | 0.014 | | -6.56 | | <0.001 | | -0.12 | | -0.07 |
| AD3xADimpprior | -0.034 | 0.020 | | -1.71 | | 0.088 | | -0.07 | | 0.01 |
| Constant | 0.918 | 0.032 | | 28.5 | | <0.001 | | 0.86 | | 0.98 |

Note: MO stands for mobility, UA for usual activities, SC for self-care, PD for pain/discomfort, and AD for anxiety/depression; “2“ denotes “some problems” and “3” denotes “severe problems”. Phase 1 stands for that the measurement was obtained during Phase 1 (within two weeks of fracture); ”…impprior” stands for reported impairment in the relevant dimension (“some problems” or “severe problems”) prior to fracture using recall.. Std stands for standard and CI for confidence internval-

1. **Intraclass correlation coefficients between value sets**

**Table S3.** Intraclass correlation estimates between value sets

|  | **ICUROS** | | |  | **Sweden EHE** | | | |  | **UK MVH** | | |
| --- | --- | --- | --- | --- | --- | --- | --- | --- | --- | --- | --- | --- |
| **Setting** | **ICC** | **95% Confidence interval** | |  | **ICC** | **95% Confidence interval** | | |  | **ICC** | **95% Confidence interval** | |
| Argentina | 0.170 | -0.079 | 0.445 |  | 0.259 | | -0.091 | 0.575 |  | 0.766 | -0.029 | 0.923 |
| Australia | 0.181 | -0.072 | 0.477 |  | 0.275 | | -0.077 | 0.613 |  | 0.832 | -0.017 | 0.951 |
| Brazil | 0.190 | -0.073 | 0.492 |  | 0.292 | | -0.085 | 0.626 |  | 0.589 | -0.063 | 0.830 |
| Canada | 0.246 | -0.092 | 0.553 |  | 0.372 | | -0.098 | 0.690 |  | 0.683 | -0.059 | 0.904 |
| Chile | 0.127 | -0.058 | 0.381 |  | 0.186 | | -0.061 | 0.497 |  | 0.928 | 0.908 | 0.943 |
| China | 0.303 | -0.081 | 0.643 |  | 0.446 | | -0.089 | 0.765 |  | 0.537 | -0.078 | 0.830 |
| Denmark | 0.188 | -0.086 | 0.460 |  | 0.290 | | -0.096 | 0.605 |  | 0.859 | 0.026 | 0.958 |
| France | 0.130 | -0.059 | 0.388 |  | 0.186 | | -0.064 | 0.494 |  | 0.935 | 0.917 | 0.950 |
| Germany | 0.175 | -0.084 | 0.439 |  | 0.254 | | -0.094 | 0.548 |  | 0.797 | -0.050 | 0.944 |
| Italy | 0.251 | -0.062 | 0.497 |  | 0.376 | | -0.014 | 0.621 |  | 0.616 | -0.065 | 0.875 |
| Japan | 0.216 | -0.090 | 0.497 |  | 0.366 | | -0.096 | 0.668 |  | 0.510 | -0.093 | 0.801 |
| Netherlands | 0.176 | -0.069 | 0.471 |  | 0.258 | | -0.074 | 0.595 |  | 0.868 | 0.087 | 0.959 |
| Poland | 0.246 | -0.084 | 0.513 |  | 0.338 | | -0.064 | 0.607 |  | 0.723 | -0.055 | 0.920 |
| Portugal | 0.096 | -0.050 | 0.312 |  | 0.143 | | -0.056 | 0.419 |  | 0.863 | 0.827 | 0.892 |
| Singapore | 0.085 | -0.046 | 0.287 |  | 0.128 | | -0.047 | 0.395 |  | 0.773 | 0.122 | 0.913 |
| South Korea | 0.276 | -0.083 | 0.550 |  | 0.465 | | -0.058 | 0.732 |  | 0.504 | -0.080 | 0.810 |
| Spain | 0.114 | -0.065 | 0.340 |  | 0.166 | | -0.074 | 0.447 |  | 0.949 | 0.935 | 0.960 |
| Sri Lanka | 0.074 | -0.056 | 0.229 |  | 0.124 | | -0.072 | 0.350 |  | 0.721 | 0.519 | 0.825 |
| Sweden | 0.737 | 0.478 | 0.850 |  | NA | | NA | NA |  | 0.179 | -0.068 | 0.477 |
| Taiwan | 0.089 | -0.034 | 0.309 |  | 0.126 | | -0.034 | 0.401 |  | 0.852 | 0.115 | 0.952 |
| Thailand | 0.110 | -0.055 | 0.343 |  | 0.166 | | -0.061 | 0.461 |  | 0.907 | 0.872 | 0.932 |
| Trinidad and Tobago | 0.290 | -0.073 | 0.557 |  | 0.454 | | -0.007 | 0.701 |  | 0.498 | -0.065 | 0.813 |
| UK | 0.123 | -0.062 | 0.369 |  | 0.179 | | -0.068 | 0.477 |  | NA | NA | NA |
| USA | 0.214 | -0.081 | 0.522 |  | 0.323 | | -0.092 | 0.653 |  | 0.685 | -0.060 | 0.905 |
| Zimbabwe | 0.329 | -0.091 | 0.661 |  | 0.442 | | -0.095 | 0.738 |  | 0.516 | -0.064 | 0.824 |
| ICUROS | NA | NA | NA |  | 0.737 | | 0.478 | 0.850 |  | 0.123 | -0.062 | 0.369 |

Note: ICC stands or intraclass correlation. The ICCs were derived from two way random effects model and pertain to the correlations between individual measurements. The command ICC in Stata 16.2 were used to derive the estimates.

**References for value sets**

The value sets are derived from the R package EQ-5D which use the following references for the EQ-5D 3L TTO value sets:

**Australia:** Viney R, Norman R, King MT, Cronin P, Street DJ, Knox S, Ratcliffe J. Time trade-off derived EQ-5D weights for Australia. Value Health. 2011 Sep-Oct;14(6):928-36

**Brazil:** Viegas Andrade M, Noronha K, Kind P, Maia AC, Miranda de Menezes R, De Barros Reis , Nepomuceno Souza M, Martins D, Gomes L, Nichele D, Calazans J, Mascarenhas T, Carvalho L, Lins C. Societal Preferences for EQ-5D Health States from a Brazilian Population Survey. Value in Health Regional Issues 2013;2(3):405–412.

**Canada:** Bansback N, Tsuchiya A, Brazier J, Anis A. Canadian valuation of EQ-5D health states: preliminary value set and considerations for future valuation studies. PLoS One. 2012;7(2):e31115.

**Chile:** Zarate V, Kind P, Valenzuela P, Vignau A, Olivares-Tirado P, Munoz A. Social valuation of EQ-5D health states: the Chilean case. Value in Health. 2011 Dec;14(8):1135-41. PubMed China: Liu GG, Wu H, Li M, Gao C, Luo N. Chinese time trade-off values for EQ-5D health states. Value Health. 2014 Jul;17(5):597-604

**Hungary:** Rencz F, Brodszky V, Gulácsi L, Golicki D, Ruzsa G, Pickard AS, Law EH, Péntek M. Parallel Valuation of the EQ-5D-3L and EQ-5D-5L by Time Trade-Off in Hungary. Value Health. 2020 Sep;23(9):1235-1245

**Poland:** Golicki D, Jakubczyk M, Niewada M, Wrona W, Busschbach JJ. Valuation of EQ-5D health states in Poland: first TTO-based social value set in Central and Eastern Europe. Value in Health. 2010;13(2):289-97

**Portugal:** Ferreira LN, Ferreira PL, Pereira LN, Oppe M. The valuation of the EQ-5D in Portugal. Qual Life Res. 2014 Mar;23(2):413-23.

**Singapore:** Luo N, Wang P, Thumboo J, Lim YW, Vrijhoef HJ. Valuation of EQ-5D-3L health states in Singapore: modeling of time trade-off values for 80 empirically observed health states. Pharmacoeconomics. 2014 May;32(5):495-507.

**Sri Lanka:** Kularatna S, Whitty JA, Johnson NW, Jayasinghe R, Scuffham PA. Valuing EQ-5D health states for Sri Lanka. Qual Life Res. 2015 Jul;24(7):1785-93.

**Sweden:** Burström K, Sun S, Gerdtham UG, Henriksson M, Johannesson M, Levin LÅ, Zethraeus N. Swedish experience-based value sets for EQ-5D health states. Qual Life Res. 2014 Mar;23(2):431- 42.

**Taiwan:** Lee HY, Hung MC, Hu FC, Chang YY, Hsieh CL, Wang JD. Estimating quality weights for EQ-5D (EuroQol-5 dimensions) health states with the time trade-off method in Taiwan. J Formos Med Assoc. 2013;112(11):699-706.

**Thailand:** Tongsiri S, Cairns J. Estimating population-based values for EQ-5D health states in Thailand. Value Health. 2011 Dec;14(8):1142-5.

**Trinidad and Tobago:** Bailey H, Stolk E, Kind P. Toward Explicit Prioritization for the Caribbean: An EQ-5D Value Set for Trinidad and Tobago. Value Health Reg Issues. 2016 Dec;11:60-67-

**Remaining countries:** Szende, A., Oppe, M., & de Charro, F. (2007), Comparative review of Time Trade-Off value sets. In Szende, A., Oppe, M., & Devlin, N. (Ed.), EQ-5D Value Sets: Inventory, Comparative Review and User Guide (pp. 27-28). Dordrecht, The Netherlands: Springer; and Janssen, B., Szende, A., & Ramos-Goñi JM. (2014), Data and Methods. Szende, A., Janssen, B., & Cabasés, J. (Ed.), In Self-Reported Population Health: An International Perspective based on EQ-5D (p 13). Dordrecht, The Netherlands: Springer.
